# *Arnica montana* Cell Culture Establishment, and Assessment of Its Cytotoxic, Antibacterial, *α*-Amylase Inhibitor, and Antioxidant In Vitro Bioactivities

**DOI:** 10.3390/plants10112300

**Published:** 2021-10-26

**Authors:** Aurelio Nieto-Trujillo, Francisco Cruz-Sosa, Rosendo Luria-Pérez, Gabriel Alfonso Gutiérrez-Rebolledo, Angélica Román-Guerrero, Cristina Burrola-Aguilar, Carmen Zepeda-Gómez, María Elena Estrada-Zúñiga

**Affiliations:** 1 Centro de Investigación en Recursos Bióticos, Facultad de Ciencias, Universidad Autónoma del Estado de México, Carretera Toluca-Ixtlahuaca Km 14.5, San Cayetano, Toluca 50295, Mexico; cba@uaemex.mx; 2 Departamento de Biotecnología, Universidad Autónoma Metropolitana-Unidad Iztapalapa, Av. Ferrocarril San Rafael Atlixco No 186, Leyes de Reforma 1ra Sección, Ciudad de México 09340, Mexico; cuhp@xanum.uam.mx (F.C.-S.); arogue@xanum.uam.mx (A.R.-G.); 3 Unidad de Investigación en Enfermedades Hemato-Oncológicas, Hospital Infantil de México Federico Gómez, Dr. Márquez No 162, Col. Doctores, Cuauhtémoc, Ciudad de México 06720, Mexico; 4 Laboratorio de Toxicología Productos Naturales, Academia de Toxicología, Departamento de Farmacia, Escuela Nacional de Ciencias Biológicas-Unidad Zacatenco, Instituto Politécnico Nacional, Av. Luis Enrique Erro S/N, Alcaldía Gustavo A. Madero, Ciudad de México 07738, Mexico; ggutierrezreb@gmail.com; 5 Facultad de Ciencias, Universidad Autónoma del Estado de México, Campus El Cerrillo, Carretera Toluca-Ixtlahuaca Km 15.5, Piedras Blancas, Toluca 50200, Mexico; zepedac@uaemex.mx

**Keywords:** Asteraceae, callus induction, flavonoids, fractionation, in vitro culture, phenolic acids, plant growth regulators, secondary metabolites, sesquiterpene lactones

## Abstract

*Arnica montana* cell suspension culture could be a sustainable source of a vegetal material producer of secondary metabolites (SMs) possessing biological effects. Different plant growth regulator concentrations (0–5 mg/L) were tested in foliar explants to induce a callus that was used to establish a cell suspension culture. Growth kinetics was carried out for 30 days. A methanolic extract obtained from biomass harvested at 30 days of growth kinetics was fractionated, and three fractions were tested for bioactivities. We induced a callus with 1 mg/L of picloram and 0.5 mg/L of kinetin in foliar explants, which allowed for the establishment of a cell suspension culture, and the latter had the highest total SMs contents at day 30. Three fractions showed differences in total SMs contents, with the highest values per gram as follows: 270 mg gallic acid equivalent for total phenolic content, 200 mg quercetin equivalent for total flavonoid content, 83 mg verbascoside equivalent for total phenolic acid content, and 396 mg parthenolide equivalent for total sesquiterpene lactone content. The best bioactivities were 2–6 µg/mL for the 50% inhibition of 2,2-diphenyl-1-picrylhydrazyl radical, 30% cellular viability of lymphoma cells at 40 µg/mL, 17% inhibition against *Escherichia coli* and *Staphylococcus aureus* at 8 µg/disk, and *α*-amylase inhibition at 12% with 10 µg/mL. The total SMs contents were correlated with bioactivities.

## 1. Introduction

*Arnica montana* L. (Asteraceae) is, globally, one of the most important medicinal plants, and for centuries, different parts of this plant have been used in ethnomedicine for many treatments, such as for osteoarthritis, bowel ache, cough, contusion, cuts, hematoma, headache, and rheumatism. Recently, research has supported these medicinal uses through scientific pharmacological and phytochemical reports [1,2,3]. This species is pharmacologically recognized for its strong anti-inflammatory activity, but it also possesses other outstanding bioactivities such as immune-modulatory, anti-sclerotic, analgesic, antifungal, antioxidant, antibacterial, and anticancer [2,3]. Over 150 biologically active substances from *A. montana* were isolated and identified, mostly belonging to terpenoids (comprising monoterpenes, essential oils, sesquiterpene lactones, diterpenes, triterpenes, and carotenoids), phenolic compounds (comprising phenolic acids, coumarins, flavonoids, and lignans), and pyrrolizidine alkaloids [3,4]. However, among them, outstanding pharmacological effects are attributed to flavonoids (such as apigenin, hispidulin, kaempferol, and quercetin) and phenolic acids (such as chlorogenic, caffeic, gallic, ferulic, and *p*-coumaric acids), which are mainly related to its antimicrobial and antioxidant activities, while sesquiterpene lactones (such as helenalin and 11*α*,13-dihydrohelenalin acetate) were mainly associated to its cytotoxic and anti-inflammatory activities [4,5,6,7,8,9]. 

*A. montana* is an herbaceous species that generally grows in nutrient-poor and dry heathlands, shrublands, and grasslands of mountainous environments. It is endemic to Europe but is also distributed in Asia and North America [10]. Wild plant populations are resources of important genetic diversity in Europe and globally. However, commercial demand for different products prepared from this medicinal plant [11] has increased the plant-material requirements of the pharmaceutical industry, leading to the overexploitation and eradication of its wild plant populations, so it is a vulnerable or threatened species with a risk of extinction [12]. Therefore, for commercial and bioethical reasons, it is important to sustainably supply the plant material [4,12,13].

Biotechnological approaches, particularly in vitro plant cultures, play a major role in researching sustainable alternatives for plant material mass production, and for developing the continuous production of biologically active SMs [14,15]. In vitro plant cultures allow for greater control than wild specimens do because they are independent of climatic and soil conditions, which leads to obtaining high yields of vegetal material or biologically active SMs. Another advantage is that they also have better quality control in terms of contamination by pollutants and phytopathogenic agents [16]. Biotechnological in vitro plant culture advances for *A. montana* mainly refer to micropropagation, where a combination of explant type, genotype, culture medium type, and plant growth regulators (PGRs) influenced the final yields of regenerated plants [17,18,19,20]. However, reports about the in vitro plant cultures of *A. montana* to produce SMs are scarce. The hairy root of this species produces thymol derivatives, flavones (chrysin), and phenolic acids (2-phenyl lactic, m-hydroxybenzoic, protocatechuic, 4-hydroxyphenyllactic, and caffeic acids) [21,22]. There are no reports about *A. montana* cell suspension culture producers of SMs, and to develop it is imperative since there was previously a significant increase in the concentration of outstanding bioactive SMs compared to wild plants: range values of 0.31% to 1.10% of total content of sesquiterpene lactones, 1.44% to 2.44% of total content of phenolic acids, and 0.6% to 1.7% of total content of flavonoids, determined in the flowers, which are the organ with the highest production of these three types of SMs in wild plants [4,23,24]. This kind of culture shows some advantages compared with in vitro organ cultures, such as fast growth, the high production of biomass, and homogeneity, to the scale-up production process in bioreactors [4,25]. Here, a cell suspension culture of *A. montana* was established, and its growth and SM production were characterized through 30 days of culture. Afterwards, the time of culture when the greatest yield of SM extraction was detected was selected to perform fractionation of the corresponding methanolic extract, followed by assessing the potential of fractions to exert cytotoxic, *α*-amylase inhibitory, antibacterial, and antioxidant activities.

## 2. Results

### 2.1. Establishment of Arnica montana Suspension Cell Culture

#### 2.1.1. Plant Growth Regulators Induced Callus and Root Formation in Foliar Explants of *A. montana*

**Figure 1:**
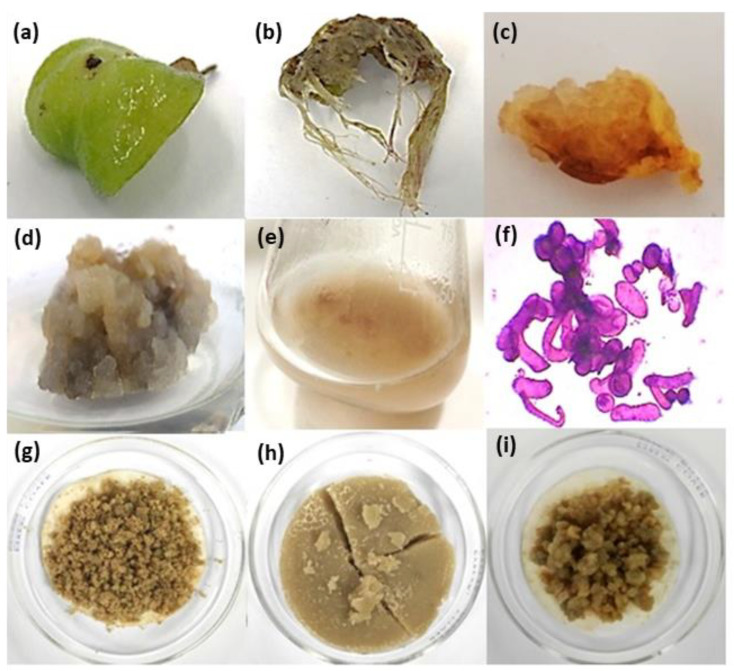
Representative callus and root cultures of *A. montana* under a supply of plant growth regulator (PGR). Effect of PGRs in foliar explants after 30 days of time culture: (**a**) control without PGRs, (**b**) root response under NAA or IBA at 1–5 mg/L, (**c**) callus induction, (**d**) callus proliferation, and (**e**) cell suspension culture and its (**f**) cells stained with safranin observed in optic microscopy at 10X under 1 mg/L of PIC with 0.5 mg/L of KIN. Harvested biomass of cell suspension cultures obtained after six subculture cycles under (**g**) 1 mg/L IBA with 1 mg/L BAP, (**h**) 1 mg/L PIC with 0.5 mg/L KIN, and (**i**) 1 mg/L PIC with 1 mg/L BAP.

To apply 0.5–5 mg/L of auxin exogenously: picloram (PIC), 2,4-dichlorophenoxyacetic acid (2,4-D), naphthaleneacetic acid (NAA), 3-indolebutyric acid (IBA), 3-indoleacetic acid (IAA), or cytokinin: 6-benzylaminopurine (BAP) and kinetin (KIN), or their combination, in foliar explants of *A. montana* significantly caused callus and root formation after 30 days of exposure, and this depended on type and concentration of tested PGR. The control treatment (without PGR addition) did not induce any response (Table 1). Comparing the influence of auxin or cytokinin on both induced responses, results showed that auxin played a major role. Among the tested auxins, PIC was a potent PGR for inducing a callus since it significantly promoted a high percentage of induction (100%), regardless of the tested concentration (0.5–5 mg/L) (Table 1). NAA at 1 to 5 mg/L simultaneously promoted callus and root formation, but a significant increase in NAA concentration increased the callus induction percentage with a decrease in the root induction percentage (Table 1). IBA was a potent PGR for inducing root formation since it significantly generated high percentages (78–100%) at 1 to 5 mg/L (Table 1). Combining auxin with cytokinin affected the percentage of callus or root induction response compared with the observed effect under only auxin or cytokinin. Combined auxin with cytokinin at a concentration lower than 2.5 mg/mL generally improved the percentages of induction, phenotypic characteristics, and callus growth, while concentrations higher than 2.5 mg/mL decreased these effects (Table 1). Moreover, the visual growth and color appearance (phenotypic characteristics) of induced callus depended on the kind and concentration of the tested PGRs (Table 1). Cultures without PGRs (control) showed foliar explants with any induction response and became chlorotic (Figure 1a). PIC, 2,4-D, and NAA, combined or not with cytokinin, generally promoted a friable callus, while IBA and IAA with cytokinin promoted a compact callus (Table 1). Root phenotypic characteristics (thin, poor branched, and with slow growth) were similar in all cases (Table 1, Figure 1b). Lastly, calluses induced by 1 mg/L of IBA with 1 mg/L of BAP, 1 mg/L of PIC with 1 mg/L of BAP, and 1 mg/L of PIC with 0.5 mg/L of KIN (Figure 1c) proliferated for three months (Figure 1d) because they showed high callus percentage induction with friable and fast visual growth. Afterwards, they were transferred into a liquid medium to establish a cell suspension culture (Figure 1e,f). During six subculture cycles, the cell suspension culture grown in 1 mg/L of PIC and 0.5 mg/L of KIN conditions continued showing friability and fast growth. When the cell suspension culture was harvested, greater biomass production was observed by comparison with other tested cell suspension culture treatments (Figure 1g–i); thus, because of its visual phenotypic stability, this cell suspension culture was selected for further experiments.

#### 2.1.2. Growth and Secondary Metabolite Total Content Characterization of Cell Suspension Culture

**Figure 2:**
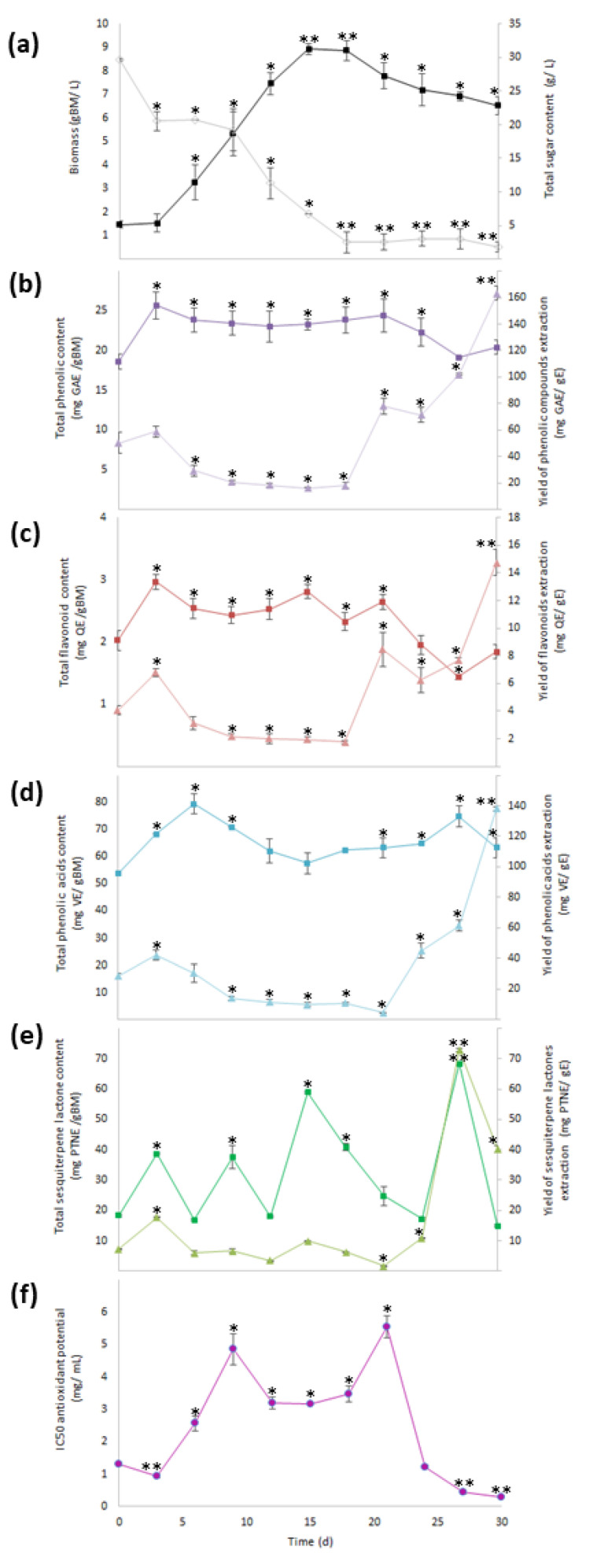
Growth and content of phenolic compounds, sesquiterpene lactones, and antioxidant activity through 30 days of culture. (**a**) Production of biomass (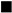) and total sugar content (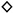), (**b**) total phenolic content (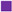) and yield of phenolics’ extraction (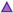), (**c**) total flavonoid content (TFC,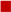 ) and yield of flavonoids’ extraction (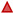), (**d**) total phenolic acid content (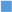) and yield of phenolic acids’ extraction (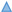), (**e**) total sesquiterpene lactone content (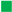) and yield of sesquiterpene lactones’ extraction (Y_SL/E_: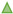 ), and (**f**) antioxidant activity (IC_50_: 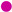). Within every line tendency, data show mean ± SD followed by *, which indicates statistical differences at the 5% level of probability regarding time culture at day 0, while ** indicates those treatments showing the highest production of biomass, total phenolic content, total flavonoid content, total phenolic acid content, total sesquiterpene lactone content, yield of phenolics’ extraction, yield of flavonoids’ extraction, yield of phenolic acids’ extraction, and yield of sesquiterpene lactones’ extraction. ** Indicates lowest values for total sugar content and antioxidant activity. All the results regarding yield of SM’s extraction were estimated with regard the corresponding total SM content per gram of extract.

During 30 days of growth kinetic evaluation, the cell suspension culture of *A. montana* showed three phases of growth: a lag phase (from 0 to 3 days), exponential phase (from 3 to 15 days), and stationary phase (from 15 to 30 days). The growth parameters were a specific growth rate of 0.145 days^−1^, doubling time of 4.8 days, growth index of 5.1, and maximal amount of dry biomass of 8.9 per gram of biomass (gBM)/L. In addition, total sugar content determined in the residual medium at day 30 was 1.8 g/L, indicating that the consumption of the carbon source was almost total compared with the total sugar content at 0 days (30 g/L) (Figure 2a).

Phytochemical analysis in the methanolic extract obtained from all dry biomass samples harvested during growth kinetics showed that SM production and antioxidant activity changed over time. The highest value for total phenolic content (22–26 mg gallic acid equivalent/gBM (mg GAE/gBM)) was determined from 3 to 24 days, with no statistical difference between them (Figure 2b), and the highest value for total flavonoid content (2.3–3.0 mg quercetin equivalent/gBM (mg QE/gBM)) was determined from 3 to 21 days as statistically the same (Figure 2c). For total phenolic acid content (63.3–79.4 mg verbascoside equivalent/gBM (mg VB/gBM)), the highest values were determined from 3 to 9 and from 21 to 30 days without statistical differences (Figure 2d). Lastly, for total sesquiterpene lactone content, the highest values (68.4 mg parthenolide equivalent/gBM (mg PTNE/gBM)) (Figure 2e) were observed at day 27.

When the total SM contents were estimated per gram of extract (yield of SMs extraction), a significant increase from 21 to 30 days was observed for total phenolic, flavonoid, and phenolic acid contents, with the highest value reached at 30 days (163 mg GAE/gE, 15 mg QE/gE, and 138 mg VBE/gE, respectively; Figure 2b–d), while the yield of extraction of total sesquiterpene lactone content had a significant increase from 24 to 30 days, with the highest value (73 mg PTNE/gE) determined at day 27 (Figure 2e).

Moreover, antioxidant activity through the inhibition of 2,2-diphenyl-1-picrylhydrazyl (DPPH) radical that had the best effect (0.3 to 0.9 mg/mL for the extract concentration required for 50% inhibition (IC_50_)) occurred in extracts obtained at days 3, 27, and 30, with all of them being statistically similar (Figure 2f). According to the results of the yield of SMs extraction, day 30 of the culture had the highest values; thus, the methanolic extract obtained from the biomass culture at day 30 was selected to perform fractionation by column chromatography.

### 2.2. Total Secondary Metabolite Contents in Fractions from Methanolic Extract 

**Figure 3:**
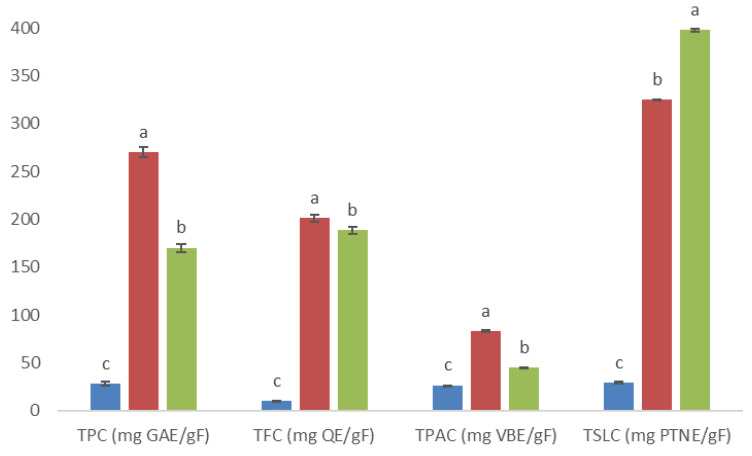
Total phenolic, flavonoid, phenolic acid, and sesquiterpene lactone contents (TPC, TFC, TPAC, and TSLC, respectively; all these results are expressed per gram of fraction) at 4AM (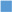), 5AM (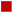), and 6AM (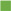) fractions obtained from a methanolic extract of cell biomass of *A. montana*. Between every secondary metabolite content, data show means ± SD followed by letters that indicate statistical differences at the 5% level of significance.

Ten fractions were collected from methanolic extract eluted through column chromatography, which showed different phytochemical profiles based on qualitative screening analysis for phenolic compounds, flavonoids, and sesquiterpene lactones (data not shown). However, only three fractions (4AM, 5AM, and 6AM) visually showed major intensity in color or precipitate positive response; thus, they were selected for testing with quantitative analysis and in vitro activity assays. Statistical analysis showed that the highest value of total phenolic content, total flavonoid content, and total phenolic acid content occurred for the 5AM fraction, while the highest value of total sesquiterpene lactone content corresponded to the 6AM fraction (Figure 3).

#### 2.2.1. Cytotoxic and Antibacterial Activities of Fractions

**Figure 4:**
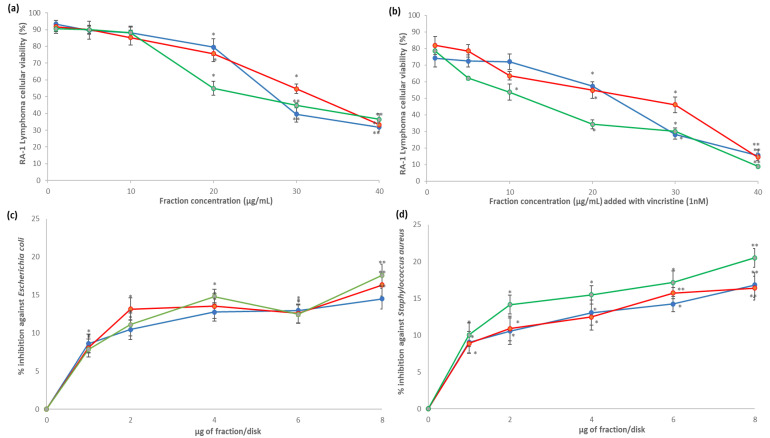
Cytotoxic and antibacterial activities of fractions obtained from the methanolic extract of cell biomass of *A. montana*. 4AM (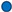), 5AM (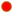), and 6AM (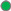) fractions showed cytotoxic effect against Ramos RA-1 lymphoma cell line (**a**) alone and (**b**) combined with 1 nM of vincristine. Antibacterial effect against (**c**) *E. coli* and (**d**) *S. aureus*. Data show means ± SD. * Indicates statistical differences at the 5% level of significance compared to the negative control, and ** indicates those treatments showing the lowest values for cellular viability of RA-1 lymphoma cells or the highest % of inhibition of bacterial growth. In cytotoxic Ramos RA-1 lymphoma cell assays, a negative control was the culture medium (which induced cellular viability of 94.71% ± 1.5%), while 1 nM of vincristine was used as a positive control (reducing cellular viability of 72.61% ± 1.45%). In the antibacterial assay against *E. coli*, the negative control was 3% DMSO, and the positive control was 1 µg chloramphenicol/disk. In the antibacterial assay against *S. aureus*, the negative control was 3% DMSO, and the positive control was 1 µg vancomycin/disk. For both antibacterial assays, data are shown as % of inhibition.

All the fractions at 20, 30, and 40 µg/mL concentrations significantly decreased the cellular viability of Ramos RA-1 lymphoma cells. However, in all cases, the greatest cytotoxic effect occurred at 40 µg/mL (almost 30% of cellular viability against 94.71% ± 1.5% was caused by the negative control; Figure 4a). With the 1 nM vincristine positive control, cellular viability was 72.61% ± 1.45%. Moreover, combining every fraction at different concentrations with 1 nM of vincristine enhanced the cytotoxic effect in cells in comparison with the observed results for fractions alone (Figure 4a,b). Under 40 µg/mL, the 6AM fraction provoked almost 7% cellular viability, while the 4AM and 5AM fractions showed 15% cellular viability (Figure 4b). Based on the extract’s concentration required for a 50% inhibition of cell growth (IC_50_) for this cytotoxic effect, the obtained values for the 4AM, 5AM, and 6AM fractions were 29.47 ± 1.14, 32.15 ± 0.60, and 28.69 ± 1.40 µg/mL, respectively (which were statistically not different comparatively). When every fraction was combined with 1 nm of vincristine, IC_50_ cytotoxic values were 19.62 ± 1.01, 21.26 ± 0.73, and 14.24 ± 1.22 µg/mL respectively, with the 6AM fraction being significantly more cytotoxic than the two other fractions (data not shown).

For antibacterial results, all fractions had significant growth inhibition against *E. coli* and *S. aureus* at all tested concentrations (1–8 µg/disk) compared to the negative control, which caused zero percentage of inhibition; however, the percentage of inhibition depended on the fraction, concentration, and bacterial strain. In the case of *E. coli*, the highest inhibition percentage was reached at 8 µg/disk for the three fractions, achieving 14.48%, 16.31%, and 17.57%, respectively (Figure 4c). For *S. aureus*, the highest inhibition percentage was reached at 8 µg/disk for the 4AM and 6AM fractions, corresponding to 16.8% and 20.48% respectively, while the highest inhibition for the 5AM fraction was at 6 and 8 µg/mL, with 15.72% and 16.39%, with both concentrations being statistically similar (Figure 4d). According to the profile of inhibition, the 6 AM fractions caused better *S. aureus* inhibition than the other two did (Figure 4d).

#### 2.2.2. Antioxidant Activity and *α*-Amylase Inhibition of Fractions

**Figure 5:**
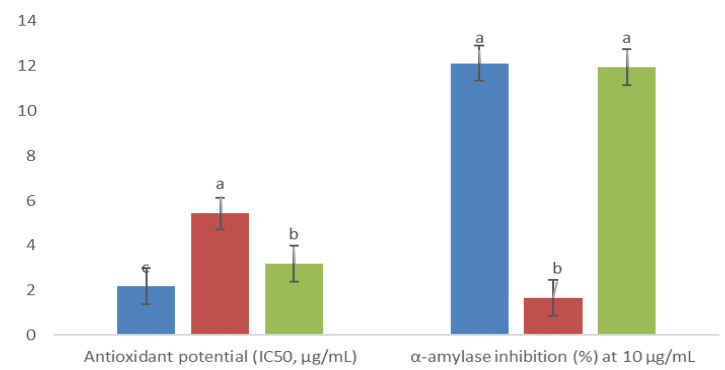
Antioxidant activity and inhibitory *α*-amylase effect of fractions obtained from methanolic extract of cell biomass of *A. montana*, 4AM (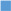), 5AM (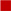), and 6AM (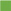). Between every effect, data show the means ± SD, and letters indicate statistical differences at the 5% level of probability.

Three fractions exhibited statistical differences in antioxidant activity, and their DPPH radical scavenging potential was as follows: 4AM > 6AM > 5AM (corresponding to IC_50_ of 2 > 3 > 6 µg/mL, respectively; Figure 5). At a 10 µg/mL concentration, all fractions inhibited *α*-amylase activity, with the better inhibition (almost 12%) achieved by the 4AM and 6AM fractions (no statistically significant difference between them; Figure 5). Comparing them with the acarbose positive control (IC_50_ of 1.83 ± 0.27 µg/mL), they showed a lower inhibitory effect.

**Figure 6:**
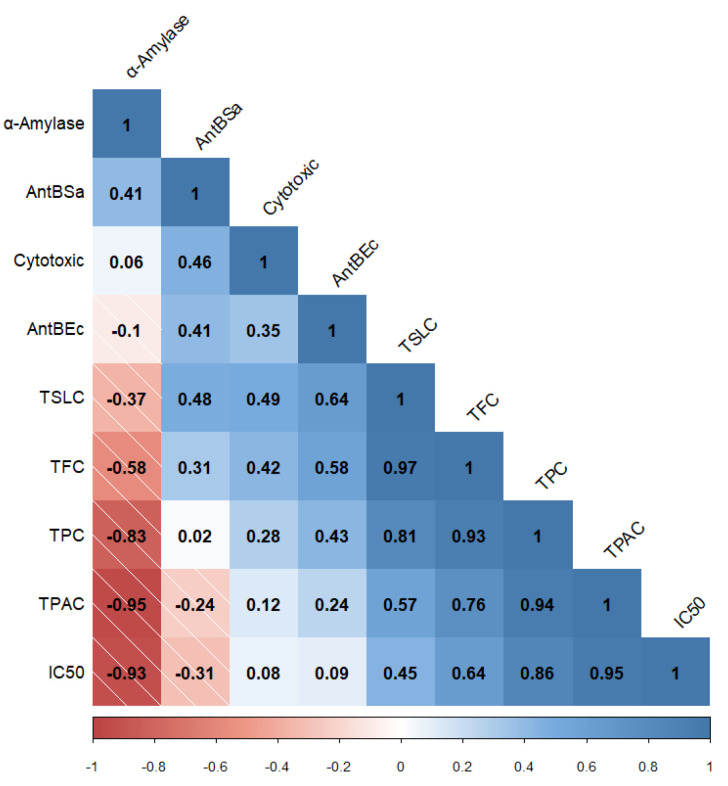
Correlogram (Pearson’s correlation) between total phenolic, flavonoid, phenolic acid, and sesquiterpene lactone contents (TPC, TFC, TPAC, and TSLC, respectively) and in vitro biological activities (*α*-amylase refers to inhibitory enzyme effect, AntBSa refers to antibacterial effect against *S. aureus*, AntBEc refers to antibacterial effect against *E. coli*, Cytotoxic refers to the cytotoxic effect on Ramos RA-1 lymphoma cells, and IC_50_ refers to antioxidant activity). The data shown correspond to statistical analysis with a 5% or lower significance level.

Lastly, since the outstanding pharmacological effects of *A. montana* are attributed to phenolic and terpenoid SMs, Pearson’s correlation analysis was conducted between the total phenolic, flavonoid, phenolic acid, and sesquiterpene lactone contents, and in vitro biological effects, tested for fractions, which allowed for establishing significant correlations (Figure 6).

## 3. Discussion

The exogenous application of auxin and cytokinin PGRs is one of the main factors that influence callus formation or morphogenetic responses in plant explants grown under in vitro conditions [26]. High concentrations of auxin generally promote rhizogenesis [27], while high amounts of cytokinins cause the induction of shoots [28], but a balance in the concentrations of both leads to callus development [27]. However, these induction responses are influenced by genotype, the endogenous concentration of PGRs, and a tissue specificity response related to the interaction between exogenous PGRs and their receptors [26]. In this work, different kinds, and concentrations of auxin and cytokinin on the foliar explants of *A. montana* plantlets influenced callus or root formation, percentage induction, and phenotypic characteristics, results that agree with those of previous studies on *A. montana*. In the apical shoots, cotyledons, hypocotyls, leaves, and petioles of *A. montana*, low concentrations of 2,4-D promote callus induction, while high concentrations cause callus necrosis [22,29,30]. Callus growth was inhibited during the second phase, adding only 2,4-D, but combining 2,4-D with BAP re-established biomass growth [30]. In cotyledon and hypocotyl explants, 1 mg/L of NAA with 0.5 and 1.0 mg/L of BAP simultaneously induced callus and root formation [30]. Combining cytokinin with auxin for callus formation is interesting for in vitro cultures, since cytokinin acts antagonistically in relation to the auxin effect in root formation [31]. Thus, since treatments here consisted of 1 mg/L of PIC with 0.5 mg/L of KIN or 1 mg/L of BAP, the induced friable calluses that showed fast growth in the leaves of *A. montana* seedlings were selected for establishing a cell suspension culture by disaggregation.

As far as our literature survey could ascertain, there are no reports or published works related to growth kinetics and SM production in cell suspension cultures of *A. montana*, and this work is the first of its kind. However, for hairy root cultures of this species, a maximal biomass of 9 g/L at day 40 was observed, with a doubling time of 7.5 days [21], which it demonstrates that doubling time is longer in roots than that observed in this work for cells in suspension, with similar production of maximum biomass. Moreover, in cell suspension cultures of species belonging to the Asteraceae family, growth parameters such as those observed in this work were reported for *A. montana*, and these cell suspension cultures were able to produce SMs. For example, in *Helianthus tuberosus* cultures, the lag phase was from day 0 to 4, the exponential phase was from day 4 to 14, and the stationary phase was from day 14 to 24, with a doubling time of 6 days and maximal biomass of 5.5 g/L [32]. For *Artemisia absintium*, the lag phase was from day 0 to 6, the exponential phase was from day 6 to 21, and the stationary phase was from day 21 to 42, with a doubling time of 9 days and maximal biomass of 9.2 g/L at day 27 [33]. For this last culture, total phenol and flavonoid contents were not strictly dependent on growth, changed through culture time for *A. absintium* cell suspension, and the highest values for total phenol and flavonoid contents were 3.6 mg GAE/gBM at day 30 and 1.9 mg QE/gBM at day 33. In the *A. absintium* cell culture, gallic and caffeic phenolic acid and catechin flavonoid production changed through time culture, but the major concentration of all these SMs was detected almost at the end of the time of culture [33]. In the *Ageratina pichinchensis* cell suspension culture, the lag phase occurred at days 0–4, the exponential phase at days 4–16, and the stationary phase at days 16–22, with a specific growth rate of 0.2 days^−1^, doubling time of 3.01 days, growth index of 5.6, and maximal biomass of 13.2 g/L at day 16. This *A. pichinchensis* cell suspension culture produced some bioactive terpenes that changed through culture time, with some reaching maximal production during the exponential or stationary phase [34]. Our results for *A. montana* coincide with those of other reported work. The presence of phenolic acids, flavonoids, and terpenoids is a common phytochemical characteristic of species belonging to the Asteraceae family [3,35]. The results of this work and the cell culture of other species belonging to the Asteraceae family demonstrate that cell cultures can synthetize characteristic SMs of this family.

Moreover, the *A. pichinchensis* cell culture showed an abrupt consumption of carbon source, almost exhausting it at 8 days of culture time [34]. In this work, *A. montana* cells almost exhausted the carbon source after ending the exponential phase. Both works emphasize that a fast consumption of the carbon source during the exponential phase probably leads to abiotic stress that influences the production of SMs during the stationary phase. Plant phenolic compounds are the most widely distributed and predominant group of SMs with substantial physiological and morphological functions. They are key defense compounds against abiotic stresses (e.g., in response to light, chilling, and pollution stimuli), and as a defense for injured plants, etc., but also play an important role in cell division, hormonal regulation, and plant growth [36,37,38]. Most plant terpenes are involved in plant defense, and a few derivatives have essential roles in plant growth and development [39,40]. Thus, due to the outstanding roles of phenolic and terpene SMs in vegetal growth and stress, they were observed in significant concentrations during both the stationary phase and the exponential phase of growth for the cell suspension cultures of *A. montana*.

**Figure 7:**
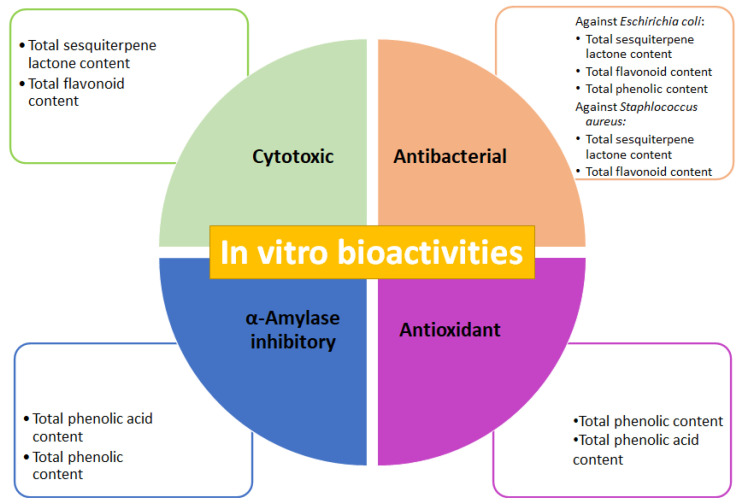
Cytotoxic, antibacterial, antioxidant, and *α*-amylase inhibitory activities of fractions obtained from methanolic extract of cell biomass of *A. montana*, and outstanding total secondary metabolites contents attributed to in vitro bioactivities.

The presence of SMs is credited with biological activities that afford plants their medicinal properties [3,35]. SMs are characterized by high structural diversity, which confers a broad spectrum of biological activities; thus, they are increasingly looked upon as a valuable natural source in the research and development of new drugs, representing novel discoveries in the health sciences [41]. To determine correlation among the total SM contents in the three fractions, isolated from methanolic complete extract of cell-dried biomass from *A. montana* cell culture, the tested biological activities (Figure 7) emphasize the possibility that these fractions contained some pharmacologically important SMs for this species. The total sesquiterpene lactone and flavonoid contents correlated to the cytotoxic effect of three fractions are consistent with the cytotoxic effect reported for *A. montana* on several cancer cell lines, such as lung carcinoma and colorectal cancer, with this effect attributed to flavonoids (mainly hispidulin, quercetin, patuletin, and jaceosidin) and helenalin, and sesquiterpene lactones (11*α*,13 dihydrohelenaliln, chamissonolide, 6-deoxychamissonolide, 2-deacetyl-4-O-tigloyl-chamissonolide), both through provoking apoptosis induction [42,43,44,45]. In addition, the fact that combining the three fractions with vincristine enhanced the cytotoxic effect on the Ramos RA-1 lymphoma cell line could be attributed to chemo-sensitization, since combining extracts from plants with a reference drug allows for decreasing the dose of the reference while simultaneously enhancing its cytotoxic effect. It was attributed to secondary metabolites from the extracts, such as phenolics and sesquiterpene lactones, which influence the signaling pathways involved in chemoresistance [46,47,48,49].

Correlation between the antibacterial effect of the three tested fractions against *E. coli* and *S. aureus,* and total sesquiterpene lactone, flavonoid, and phenolic contents, was determined, agreeing with previously published reports, such as those results of an aqueous extract of wild *A. montana* flowers, which provoked a significant antibacterial effect against *E. coli* and *S. aureus*, with a minimal inhibitory concentration value of 16.7 mg/mL for both strains. This antibacterial activity showed a positive correlation with total phenolic content [50]. Ethanolic and methanolic extracts of wild *A. montana* flowers have an antibacterial effect against methicillin-resistant *S. aureus* (MRSA) [51] and extracts reduced biofilm formation by 42.3% [52].

The fact that the *α*-amylase inhibitory effect of the three fractions was negatively correlated to total phenolic and phenolic acid contents was probably provoked by the presence of a phenolic acid compound showing a great capacity to inhibit this enzyme, despite its concentration not being very high. In some other species of the Asteraceae family, phenolic compounds, and particularly phenolic acids act as *α*-amylase inhibitors. The ethanolic extract of *Artemisia commutata* (Asteraceae) presented the highest *α*-amylase inhibition (IC_50_ = 150.24 µg/mL) regarding several species belonging to the *Artemisia* genus (IC_50_ 207.12 µg/mL). This effect was positively correlated to total phenolic acid content (514.65 ± 15.43 mg/g in *A. commutata*) and weakly correlated with total flavonoid content [53]. Leaves or roots of methanolic extracts of *Achyranthes aspera*, *Eclipta alba*, and *Vitex negundo* (all belonging to the Asteraceae family) had an inhibitory effect on *α*-amylase activity, showing that leaf extracts had a better effect than that of root extracts. Among species, *V. negundo* showed the greatest inhibition (70.95% at 0.25 mg/mL). Those results showed significant correlation between enzyme activity inhibition, and total phenolic and flavonoid contents [54]. In *Cota fulvida* (Asteraceae), the methanolic extract of its aerial parts demonstrated significant inhibition of *α*-amylase with an IC_50_ of 0.35 mg/mL, an effect attributed to flavonoid concentration in the extract [55]. Phenolic acids in species belonging to the Asteraceae family showed capability to inhibit *α*-amylase, whose IC_50_ was reported in mg/mL (6.0 mg/mL for syringic acid, 0.5 mg/mL for chlorogenic acid, 1.8 mg/mL for salicylic acid, 3.5 mg/mL for caffeic acid, 6.2 mg/mL for vanillic acid, 5.6 mg/mL for *p*-coumaric acid, and 8.3 mg/mL for sinapic acid) [56]. In this work, the fractions of the methanolic extract of *A. montana* cell culture showed inhibition of this enzyme of almost 10% at 10 µg/mL. Phenolic acids could be used for the treatment of diabetes mellitus since they can alter the absorption of glucose via the inhibition of carbohydrate-hydrolyzing enzymes, such as *α*-amylase, because the inactivation of this enzyme is an option in hyperglycemia prevention [57].

Lastly, the high positive correlation between total phenolic and phenolic acid contents with an antioxidant activity of three fractions isolated from the methanolic extract of *A. montana* cell culture was consistent with previously published reports, which supports that most phenolic compounds exert antioxidant activity due to their chemical structure [58,59]. The ethanolic and water-soluble extracts of *A. montana* flowers had IC_50_ values of 0.66 and 1.71 mg/mL respectively, for antioxidant activity [60]. The flower methanolic extract of *Acmella ciliate* (Asteraceae) showed a positive correlation between antioxidant activity and total phenolic and flavonoid contents, which has also been reported in other species [61]. The fact that antioxidant activity was greatly increased in the three fractions by comparison to the complete methanolic extract of cell-dried biomass of *A. montana* (almost 50- to 150-fold), which was even higher than that reported in flower extracts [60], highlights the potential of a cell culture of *A. montana* as a source of antioxidant compounds, and the importance of performing fractionation that allows for concentrated SMs, thus improving bioactivities. Future research must be carried out to isolate, identify, and quantify bioactive compounds produced by established cell cultures of *A. montana*, responsible for the in vitro tested biological activities; additionally, future research must also be conducted to determine bioactivities using in vivo experimental models related to previously evaluated biological activities. Lastly, since *A. montana* is a species rich in phenolic acids, flavonoids, and sesquiterpene lactones with high pharmaceutical value [62], the establishment of a cell suspension culture with fast growth and sustainable production of SMs, mostly those related to the high pharmaceutical and medical value, could represent the basis for further biotechnological advances and studies of this species without undermining natural resources.

## 4. Materials and Methods

### 4.1. Plant Material and Aseptic Culture

*Arnica montana* seeds were purchased from Hortaflor^®^ (www.rancholosmolinos.com, accessed on 20 October 2021). Seeds (50–70 units) were placed inside a filter paper bag and washed for 15 min in a solution of detergent (ROMA©, Ecatepec, México). Then, the bag was sequentially immersed for sterilization under low shaking in an antibiotic solution (200 mg/L ampicillin, 200 mg/L erythromycin, and 200 mg/L of tetracycline) for 30 min, fungicide solution (10 g/L of Fungoxyl® and 10 mL/L of Bravo 720^®^, San Luis Potosí, México) for 30 min, 70% ethanol solution (*v*/*v*) for 15 s, and 20% (*v*/*v*) chlorine solution for 15 min. Lastly, under aseptic conditions, the bag was rinsed 4 times with sterile distilled water and cut, and seeds were placed inside glass bottles containing 20 mL of culture medium. Five seeds were inoculated per bottle in triplicate (n = 15) and incubated for 30 days, where germination and seedling development took place. Afterwards, the resulting plantlets were used as a source of foliar explants, which were removed from all plantlets and transferred to a culture medium supplied with PGRs.

### 4.2. Culture Medium and Incubation Conditions

A free PGR culture medium was used for the germination of seeds, which consisted of half-strength Murashige and Skoog culture medium [63], supplemented with 30 g/L glucose, 150 mg/L ascorbic acids, and 100 mg/L citric acids. This basal formulation was used for all the experiments and was prepared with distilled water. Moreover, when a semisolid culture was required (for germination, testing effect of PGRs and subculture of callus cultures), this culture medium was added with 3 g/L of phytagel, and a liquid culture was prepared without phytagel. Once all components of the culture medium had been mixed, the pH value was adjusted at 5.7–5.8, and it was autoclave-sterilized at 121 °C for 18 min. Erlenmeyer flasks containing liquid culture medium at one-fifth of the total volume were used to establish cell suspension cell culture and growth kinetics experiments; once inoculated, they were put on an orbital shaker at 110 rpm. All cultures were incubated in a 16 h photoperiod of white fluorescent light/8 h darkness, 50 µmol m^−2^ s^−1^, and 25 ± 2 °C.

### 4.3. Effect of PGRs in Foliar Explants and Callus Proliferation

Foliar explants (n = 3) were inoculated per glass bottle containing 20 mL of basal culture medium added with a combination of auxins: 2,4-D, NAA, IAA, IBA, or PIC, with cytokinins: BAP or KIN, and both kinds of PGRs were mixed up at concentrations of 0, 0.5, 1.0, 2.5, and 5.0 mg/L. Free PGR culture medium was used as a control. Resulting cultures were incubated for 30 days—the time of culture where induction percentage was determined as a ratio between explants that showed callus or morphogenetic response about the total tested explants. Every treatment consisted of two bottles, in duplicate. Calluses showing the best growth, friability, and high induction percentage were sub-cultured to proliferate biomass for three months in the same culture medium conditions used for callus induction, and every subculture cycle was every 3 weeks.

### 4.4. Cell Suspension Culture Establishment and Growth Kinetics

Proliferated callus was used as inoculum (3 g of fresh callus/50 mL of culture medium) of Erlenmeyer flasks containing liquid culture medium (same formulation used for callus induction). Resulting suspension cultures were incubated for 15 days following their subculture. Six subculture cycles were performed to screen phenotypic characteristics of the culture (friability, visual growth, color appearance), and to obtain enough biomass for use in growth kinetics experiments; every 2 subculture cycles, the volume of the Erlenmeyer flask was changed, beginning with 250 until 1000 mL (ratio of 3 g of fresh callus/50 mL of culture medium kept as inocula). A cell suspension culture showing unchanged phenotypic characteristics was selected to carry out growth kinetics for 30 days, inoculating 1.5 g of fresh cells in 125 mL Erlenmeyer flasks containing 25 mL of culture medium. Sampling was carried out every 3 days through vacuum filtration, rinsing the resulting biomass with distilled water, and drying in an oven for 24 h at 45 °C. Dried biomass was also weighed. A sample of the culture medium was recovered for analyzing TSC, employing the phenol sulfuric acid method [64]. This parameter was used as a measure of carbon source consumption by the cultured cells while kinetics lasted. Each point of growth kinetics consisted of 3 experimental units in triplicate (n = 3). Growth parameters (specific growth rate, doubling time, and growth index) of the cell suspension culture were estimated according to Arano-Varela et al. [65]. Moreover, the duration of the growth phases was determined by plotting the natural logarithm of cell growth data versus time.

### 4.5. Analysis of Secondary Metabolites’ Production

#### 4.5.1. Obtaining Methanolic Extract from Cell Biomass 

Dried cell biomass (100 mg), recovered at every sampling of the growth kinetics, was extracted with MeOH (125 mL) for 30 min in an ultrasonic homogenizer (SIJA LAB, model SJIA-950 W, Cixi, China) programmed at 20 kHz, 50 °C, with on/off cycles of 9 and 6 s, respectively. The extracts were concentrated on a rotary evaporator until dry. The dried extracts were weighed to calculate the final yield, and a sample of this extract was used to carry out phytochemical analysis.

#### 4.5.2. Quantification of Phenolic Compounds and Sesquiterpene Lactones

TPC, TFC, and TPAC were determined according to Vazquez-Marquez et al. [66]. These spectrophotometric methods are common in phytochemical analysis to determine the content of secondary metabolites based on the use of reference standards for comparison [67,68,69]. For TPC, a gallic acid standard (Sigma-Aldrich, St. Louis, MO, USA) was used to prepare solutions at concentrations from 6 to 100 µg/mL to build the calibration curve (y = 8.2506x – 0.0048, R^2^ = 0.9995). TPC results are expressed as milligrams of gallic acid equivalents per gram of biomass (mg GAE/gBM). Moreover, results are expressed as milligrams of gallic acid equivalents per gram of extract (mg GAE/gE) to determine the yield of extraction of phenolic compounds. TFC had some modifications. Briefly, a volume of 1.5 mL of 2% (*v*/*v*) AlCl_3_ in MeOH was mixed with 1.5 mL of extract, standard or MeOH (control), incubated for 10 min under darkness at room temperature, and used to measure absorbance at 415 nm. Quercetin standard (Sigma-Aldrich, St. Louis, MO, USA) was used to prepare solutions at concentrations from 0.35 to 100 µg/mL, used to build the calibration curve (y = 10.612x – 0.004, R^2^ = 0.9919). TFC results were expressed as milligrams of quercetin equivalents per gram of biomass (mg QE/gBM). In addition, results were expressed as milligrams of quercetin equivalents per gram of extract (mg QE/gE) to determine the yield of extraction of flavonoids. TPAC results are expressed as milligrams of verbascoside equivalents per gram of biomass (mg VBE/gBM). Verbascoside standard (Sigma-Aldrich) was used to prepare solutions at concentrations from 0.16 to 2.5 mg/mL, used to build the calibration curve (y = 0.7508x + 0.0449, R^2^ = 0.9985). Additionally, results were expressed as milligrams of verbascoside equivalents per gram of extract (mg VBE/gE) to determine the yield of extraction of phenolic compounds. Lastly, TSLC was executed according to Salapovic et al. [70], using parthenolide as a standard. It was prepared at different concentrations (4.96–19.86 µg/mL) to build the calibration curve (y = −0.0008x + 0.1743, R^2^ = 0.9757). Salapovic et al. validated a spectrophotometric method to determine the total sesquiterpene lactone content in extracts of different species belonging to the Asteraceae family. TSLC results were expressed as milligrams of parthenolide equivalents per gram of biomass (mg PTNE/gBM). Furthermore, results were also expressed as milligrams of parthenolide equivalents per gram of extract (mg PTNE/gE) to determine the yield of extraction of sesquiterpene lactones. In all cases, absorbance measurements were carried out in a UV-vis spectrophotometer (Thermo Scientific, model evolution 60S, Waltham, MA, USA). Every measurement was carried out in triplicate (n = 3).

#### 4.5.3. Determination of Antioxidant Activity

IC_50_ of DPPH radical was calculated for the complete methanolic extract and its fractions and was determined to assess its antioxidant activity. This assay is a common method used for estimating the efficiency of substances as antioxidants based on the scavenging of the stable free DPPH radical [71]. Briefly, DPPH standard (Sigma-Aldrich) was used to prepare a methanolic solution at 0.1 mM, while the dried extract was reconstituted in MeOH for preparing different concentrations (0.1–10 mg/mL). A volume of 1.8 mL of 0.1 mM DPPH was mixed with 0.3 mL of the different concentrations of the complete extract or MeOH, incubated for 15 min under darkness at room temperature; then, an absorbance measurement at 515 nm was carried out in a UV-vis spectrophotometer (Thermo Scientific, model evolution 60S). Every measurement was carried in triplicate (n = 3). Results are expressed as an amount in mg/mL of the extract capable to inhibit the DPPH radical at 50%. The IC_50_ was calculated using the equation of the line, where x was found, and the value of y = 50.

### 4.6. Fractionation of Methanolic Extract and Phytochemical Analysis of Resulting Fractions

Extracts were produced, as mentioned above, using 500 mL of methanol for every 3 g of dried cell biomass harvested at 30 days of culture. Fractionation was carried out with column chromatography using a silica gel mesh size of 60–200 as the stationary phase and increasing polarity through mixing (A) acetonitrile and (B) methanol. A total of 10 fractions were obtained with a volume of 100 mL each. Resulting fractions were concentrated in a rotary evaporator and brought to total dryness in an oven at 60 °C for 24 h, and dried fractions were stored under refrigerator conditions (4 °C). Qualitative tests for Baljet, AlCl_3_, and sodium hydroxide (KOH) [72] were carried out for screening sesquiterpene lactones, phenolic compounds, and flavonoids respectively, in different obtained fractions. Those fractions that had great visual results (4AM, 5AM, and 6AM) were selected for quantitative analysis and to determine the antioxidant as mentioned above, but the results are expressed per gram of fraction. These selected fractions were used for further experiments about biological activities. In the Baljet test, 100 µL of sample and three drops of the reagent are added, and the test is positive if it acquires orange or dark red coloration. In the ferric chloride test, three drops of reagent (5% in ethanol) were added to 100 µL of sample, and the appearance of a red, blue-violet, or green precipitate was considered positive for phenolic compounds. For the sodium hydroxide test (10%), three drops of reagent were added to 100 µL of sample, and the appearance of yellow or orange was indicative of the presence of flavonoids. A greater intensity of coloration or precipitate is related to a greater concentration of SMs [72].

### 4.7. In Vitro Bioactivities of Fractions

#### 4.7.1. Cytotoxicity Assay

The human B non-Hodgkin lymphoma (Burkitt) cell line (Ramos RA-1) was obtained from the American Type Cell Collection (ATCC, CRL-1596), and cultured in RPMI 1640 Advanced medium (Invitrogen^®^, Waltham, MA, USA), supplemented at 5% with fetal bovine serum (FBS, Invitrogen^®^, Waltham, MA, USA), 1% antibiotic–antifungal containing 10,000 U/mL of penicillin G, 10 mg/mL of streptomycin, and 25 μg/mL of amphotericin B. Cultures were kept at 37 °C and 5% CO_2_. For the cytotoxicity assay, cultures were supplemented with 2% FBS.

The exclusion staining method was performed using trypan blue, and 50,000 cells per well were cultured in 96-well plates with Advanced-RPMI 1640 medium. Untreated cells were placed as a negative control, 1 nM vincristine was used as a positive control, and treatments consisted of previous selected fractions at concentrations of 1, 5, 10, 20, 30, and 40 µg/mL previously dissolved in 0.01% DMSO. Cells were treated for 24 h with fractions alone or in combination with 1 nM of vincristine (which was added 6 h before completing the 24 h of incubation), and plates were incubated at 37 °C and 5% CO_2_. Volume of cells used per well was 50 µL, and the rest was culture medium and treatments, having 100 µL as the final volume per well. To count cells after treatments, 20 µL was taken from each well, previously resuspended, and mixed with 80 µL of trypan blue; then, 10 µL was placed on each side of a Neubauer chamber and proceeded to count viable cells on an inverted microscope with a 40x objective. Resulting data are provided as a percentage of cellular viability under the following formula:

$$Cellular\;viability\;\left(\%\right)=\frac{\left(Number\;of\;total\;cells-Number\;of\;dead\;cells\right)}{Number\;of\;total\;cells}*100$$

#### 4.7.2. Antibacterial Activity Assay of Fractions

The methodology was based on previous work by Echeverría et al. [73] with some modifications. The in vitro test consists of the Kirby–Bauer method against *Staphylococcus aureus* (ATCC 25923) and *Escherichia coli* (ATCC 25922) strains. Microorganism inoculum was prepared in 5 mL of brain–heart infusion broth, adjusting to 0.5 on the McFarland scale, equivalent to 1 × 10^6^ CFU/mL, incubated at 37 °C for 24 h. Subsequently, 80 µL of inoculum was spread onto Muller Hinton agar plates using an L-shaped plastic rod, and filter paper disks impregnated with 10 µL of each fraction were placed. Vancomycin (1 µg/disk) was used as a positive inhibition control for *S. aureus* and chloramphenicol (1 µg/disk) as a positive inhibition control for *E. coli*, while fraction diluent was used as a negative control, and sterile water as a growth control. Plates were incubated at 37 °C for 24 h, during which time the inhibition halo caused by all controls was measured with the help of a Vernier caliper. The above selected fractions were dissolved in 3% DMSO and prepared at concentrations of 100, 200, 400, 600, and 800 µg/mL (1, 2, 4, 6, and 8 µg/disk). This experiment was performed in seven repetitions in triplicate for each concentration against each bacterial strain (n = 21). Data are shown as a percentage of inhibition under the following formula:

$$\%\;inhibition=\frac{\left(ZHI\;of\;fraction-ZHI\;of\;negative\;control\right)}{(ZHI\;of\;positive\;control-ZHI\;of\;negative\;control}*100$$

where ZHI corresponds to zone halo inhibition.

#### 4.7.3. α-Amylase Inhibitory Activity

The methodology proposed by Jimoh [74] was used, with some modifications. Five variables were carried out, designated as AM, AE, AEC, AMC1, and AMC2. The AM variable consisted of 250 µL of porcine *α*-amylase enzyme (2U dissolved in PBS) mixed with 150 µL of buffer plus 100 µL of fraction sample, incubated for 20 min at 37 °C. Subsequently, 500 µL of starch solubilized in water at 0.5% was added and left to rest for 20 min at 37 °C; then, 500 µL of dinitrosalicylic acid (DNSA) was added, and tubes were boiled for 15 min. The reaction stopped by immersing tubes in ice water. Lastly, the mixture was diluted with 5 mL of distilled water, and absorbance at 540 nm was measured in a UV-vis spectrophotometer, using water as a blank. Variable AE consisted of adding enzyme plus water, starch, and DNSA, AEC consisted of adding enzyme plus water, buffer instead of starch, and DNSA, AMC1 consisted of adding enzyme plus sample, buffer, and DNSA, and AMC2 consisted of adding enzyme plus sample, starch, and water instead of DNSA. Fraction concentrations of 0.625, 1.25, 2.5, 5, 7.5, 10, 20, and 50 µg/mL were tested. A concentration of 1 µg/mL of acarbose was used as a positive control, while water was the negative control. Information processing was carried out as mentioned below: *a* = Absorbance _AM_ ‒ (Absorbance _AMC1_ ‒ Absorbance _AMC2_); *b* = Absorbance _AE_ ‒ Absorbance _AEC_. Data are shown as a percentage of inhibition according to the following formula:

$$\%\;inhibition=\frac{\left(b-a\right)}{b}*100$$

### 4.8. Statistical Analysis

All the results about callus and root induction percentage, total secondary metabolite contents, yield of extraction of secondary metabolite contents, inhibition percentage for bacteria and *α*-amylase, percentage of viability in RA-1, and IC_50_ for antioxidant activity were statistically analyzed through ANOVA, followed by a Tukey–Kramer test. Pearson’s correlation (R) determined the correlation among total secondary metabolite contents and biological activities (cytotoxic, antibacterial, *α*-amylase inhibitor, and antioxidant). RStudio was used to build a correlogram. NCSS software (2007 version) was used for all the analyses, and *p* < 0.05 was assumed to indicate significant differences in all statistical analyses.

## 5. Conclusions

Picloram induces callus formation in foliar explants of *A. montana*, and its combination with kinetin (1 and 0.5 mg/L, respectively) allows for establishing a cell suspension culture showing friability, fast growth, and production of phenolic compounds (such as phenolic acids and flavonoids), terpenoids (such as sesquiterpene lactones), and secondary metabolites related to the pharmaceutical value of this species. Three fractions from the methanolic extract showed differences in their total content of secondary metabolites, and exerted cytotoxic, antibacterial, *α*-amylase inhibition, and antioxidant activities. The cell culture established in this work represents the beginning of the development of a sustainable source of a vegetal material producer of compounds possessing biological effects, without affecting the wild populations of this species.

## Figures and Tables

**Table 1:** Effect of different auxin and cytokinin plant growth regulators at several concentrations in foliar explants of *A. montana* after 30 days.

Type of Plant Growth Regulator (PGR) (mg/L)							Type of Induced Response				
							Callus				Root
Auxin					Cytokinin		Induction (%)	Phenotypic Characteristics			Induction (%)
PIC	2,4-D	NAA	IBA	IAA	BAP	KIN		Texture	Color	Growth	
Control treatment (without PGRs)							0 ± 0 ^a^				0 ± 0 ^a^
0.5							100 ± 0 ^f^	F	W; PY	+++	0 ± 0 ^a^
1							100 ± 0 ^f^	F	W; PY	++	0 ± 0 ^a^
2.5							100 ± 0 ^f^	F	W; PY	++	0 ± 0 ^a^
5							100 ± 0 ^f^	F	W; PY	+	0 ± 0 ^a^
	1						78 ± 0 ^e^	C	B	+	0 ± 0 ^a^
		1					0 ± 0 ^a^				100 ± 0 ^e^
		2.5					50 ± 0 ^c^	F	G	++	50 ± 0 ^c^
		5					75 ± 0 ^e^	F	W	+	25 ± 0 ^b^
			1				0 ± 0 ^a^				100 ± 0 ^e^
			2.5				0 ± 0 ^a^				100 ± 0 ^e^
			5				0 ± 0 ^a^				78 ± 0 ^d^
					0.5		0 ± 0 ^a^				25 ± 0 ^b^
0.5					0.5		100 ± 0 ^f^	F	Y	++	0 ± 0 ^a^
1					0.5		100 ± 0 ^f^	F	Y	+++	0 ± 0 ^a^
0.5					1		100 ± 0 ^f^	F	Y	++	0 ± 0 ^a^
1					1		100 ± 0 ^f^	F	W	+	0 ± 0 ^a^
2.5					2.5		56 ± 19 ^cd^	C	W	+	0 ± 0 ^a^
0.5						0.5	100 ± 0 ^f^	F	Y	++	0 ± 0 ^a^
1						0.5	100 ± 0 ^f^	F	Y	+++	0 ± 0 ^a^
0.5						1	100 ± 0 ^f^	F	Y	++	0 ± 0 ^a^
1						1	78 ± 19 ^e^	C	W	+	0 ± 0 ^a^
2.5						2.5	33 ± 0 ^b^	C	W	+	0 ± 0 ^a^
	1				1		100 ± 0 ^f^	F	PB	+	0 ± 0 ^a^
	1					1	100 ± 0 ^f^	F	B	+	0 ± 0 ^a^
		1			1		56 ± 19 ^cd^	F	PG	+	56 ± 19 ^c^
		2.5			2.5		33 ± 0 ^b^	F	PG	++	78 ± 19 ^d^
		5			5		56 ± 19 ^cd^	C	PB	+	33 ± 0 ^b^
		1				1	0 ± 0 ^a^				56 ± 19 ^c^
		2.5				2.5	0 ± 0 ^a^				56 ± 19 ^c^
		5				5	0 ± 0 ^a^				56 ± 19 ^c^
			0.5		0.5		0 ± 0 ^a^				100 ± 0 ^e^
			1		0.5		78 ± 19 ^e^	C	PY	+	33 ± 0 ^b^
			0.5		1		100 ± 0 ^f^	C	Y	++	0 ± 0 ^a^
			1		1		44 ± 19 ^bc^	C	Y	+++	33 ± 0 ^b^
			0.5			0.5	56 ± 19 ^cd^	C	PB	+	33 ± 0 ^b^
			1			0.5	0 ± 0 ^a^				100 ± 0 ^e^
			0.5			1	0 ± 0 ^a^				100 ± 0 ^e^
			1			1	0 ± 0 ^a^				100 ± 0 ^e^
			2.5			2.5	0 ± 0 ^a^				100 ± 0 ^e^
			5			5	33 ± 0 ^b^	C	B	++	33 ± 0 ^b^
				1	1		33 ± 0 ^b^	C	DB	+	33 ± 0 ^b^
				2.5	2.5		44 ± 19 ^bc^	C	G	+	0 ± 0 ^a^
				1		1	33 ± 0 ^b^	C	DG	+	33 ± 0 ^b^
				2.5		2.5	0 ± 0 ^a^				33 ± 0 ^b^
				5		5	0 ± 0 ^a^				78 ± 19 ^d^

Treatments able to induce callus or root. Within induction column (%), data show mean ± SD, followed by lowercase letters that indicated statistical differences at the 5% level of significance compared to the control. Abbreviations: C = compact; F = friable; B = brown; DB = dark brown; DG = dark green; G = green; PB = pale brown; PG = pale green; PY = pale yellow; W = white; Y = yellow. Growth visually observed with low intensity (+), middle intensity (++), and high intensity (+++).

## Data Availability

Not applicable.
